# Biochemical characterisation of a Kunitz-type inhibitor from *Tamarindus indica* L. seeds and its efficacy in reducing plasma leptin in an experimental model of obesity

**DOI:** 10.1080/14756366.2017.1419220

**Published:** 2018-01-11

**Authors:** Amanda Fernandes de Medeiros, Izael de Sousa Costa, Fabiana Maria Coimbra de Carvalho, Sumika Kiyota, Beatriz Blenda Pinheiro de Souza, Daniel Nogoceke Sifuentes, Raphael Paschoal Serquiz, Bruna Leal Lima Maciel, Adriana Ferreira Uchôa, Elizeu Antunes dos Santos, Ana Heloneida de Araújo Morais

**Affiliations:** a Postgraduate Biochemistry Program, Biosciences Center, Federal University of Rio Grande do Norte, Natal, Brazil;; b Laboratory of Protein and Peptide Biochemistry, CPDSA, Biological Institute, São Paulo, Brazil;; c Postgraduate Biological Molecular, Institute of Biological Sciences, University of Brasília, Distrito Federal, Brasília, Brazil;; d Embrapa Genetic Resources and Biotechnology, Embrapa, Distrito Federal, Brasília, Brazil;; e Postgraduate Nutrition Program, Center for Health Sciences, Federal University of Rio Grande do Norte, Natal, Brazil;; f Department of Nutrition, Center for Health Sciences, Federal University of Rio Grande do Norte, Natal, Brazil;; g Department of Cell Biology and Genetics, Biosciences Center, Federal University of Rio Grande do Norte, Natal, Brazil;; h Department of Biochemistry, Biosciences Center, Federal University of Rio Grande do Norte, Natal, Brazil

**Keywords:** Tamarind, antitryptic, Wistar rats, CCK

## Abstract

A trypsin inhibitor isolated from tamarind seed (TTI) has satietogenic effects in animals, increasing the cholecystokinin (CCK) in eutrophy and reducing leptin in obesity. We purified TTI (pTTI), characterised, and observed its effect upon CCK and leptin in obese *Wistar* rats. By HPLC, and after amplification of resolution, two protein fractions were observed: Fr1 and Fr2, with average mass of [M + 14H]^+^ = 19,594,690 Da and [M + 13H]^+^ = 19,578,266 Da, respectively. The protein fractions showed 54 and 53 amino acid residues with the same sequence. pTTI presented resistance to temperature and pH variations; IC_50_ was 2.7 × 10^−10 ^mol.L^−1^ and Ki was 2.9 × 10^−11 ^mol.L^−1^. The 2-DE revealed spots with isoelectric points between pH 5 and 6, and one near pH 8. pTTI action on leptin decrease was confirmed. We conclude that pTTI is a Kunitz trypsin inhibitor with possible biotechnological health-related application.

## Introduction

Obesity is a chronic non-communicable disease (CNCD) with a prevalence that has more than doubled since the 1980s^1^. By 2015, a total of 603,700 million adults and 107,700 million children were obese^2^. There is evidence that individuals and animals with obesity do not show the same sensitivity to the satietogenic effects of cholecystokinin (CCK) when compared to eutrophic, and the resistance to leptin is increased especially in the state of obesity^3–5^.

CCK is a regulatory hormone of the centre of hunger and satiety. This regulation involves the stimulation of insulin and glucagon secretion, and induction of satiety is given via gastric receptors that carry information to the hypothalamus and the nucleus of the solitary tract via the vagal afferent nerve^6^. There are also reports of its interaction with leptin, related to appetite and hunger control^3^. Leptin is an adipokine secreted hormone in proportion to the amount of adipose tissue and its concentrations increase with caloric intake and decrease during the fasting period, being elevated in obesity^7^.

Several studies have shown the satietogenic ability of trypsin inhibitors isolated from different sources, stimulating CCK increases^8–12^. Thus, these CCK stimulators are commonly called CCK secretagogues^9–11^^,^^13^^,^^14^.

Peptidase inhibitors, which include trypsin inhibitors, act as natural regulators of proteolytic enzymes^15^ and are therefore targets for research with potential biotechnological applications in a number of areas, such as medicine and agriculture^16^. Trypsin inhibitors are often found in seeds, and many of these proteins have been purified and characterised^17^.

A thorough biochemical and functional characterisation are possible after protein purification. Data such as sequencing, determination of three-dimensional structures, inhibitory specificity for classes of peptidases and inhibition mechanisms are essential^18–20^ and widely used in the analysis of substances of bioanalytical, alimentary, and pharmaceutical importance^21^^,^^22^.

The seeds from the tamarind fruit (*Tamarindus indica* L.) are source of different trypsin inhibitors. However, the trypsin inhibitors of this seed are still poorly studied, and some studies present the bioinsecticidal, anti-inflammatory, and satietogenic properties of these molecules^11^^,^^12^^,^^23–26^.

A previous study with the partially purified trypsin inhibitor from tamarind seeds (TTI) evaluated CCK and leptin concentrations in obese Wistar rats and found that TTI did not increase plasma CCK, but decreased plasma leptin^25^. Results presented by Ribeiro et al.^11^, also using TTI, suggest a synergistic action of TTI on these two hormones, depending on the condition of nutritional status. However, in these studies, TTI was only partially purified and we cannot guarantee which molecule(s) is/are responsible for the observed effects. Thus, in the present study we purified, characterised, and partially sequenced the trypsin inhibitor of the tamarind seed, reevaluating its effect upon plasmatic CCK and leptin in an experimental model of obesity. This is a necessary step for a promising protein isolate for bioprospecting new bioproducts and necessary to ascertain its action as a pure molecule.

## Materials and methods

### Plant material

The tamarind fruit, from the *Tamarindus* genus and *indica* L. species, which belongs to the Fabaceae family^27^ was obtained, and botanically identified by the IBAMA (Environmental- middle Brazilian Institute) seed bank in Natal-RN, Brazil. The pulp was separated from the seed, and the flour from the peeled seeds was used for the experiments.

### Animals

Male, obese (350–400 g), according to Lee index (>0.300 g/cm³)^28^. Wistar rats with age of 17 weeks, were provided by Potiguar University vivarium and kept in the laboratory under standard light–dark cycle conditions (12/12 h), temperature between 23 and 25 °C, water and food *ad libitum*.

The research was conducted according to Guide for the Care and Use of Laboratory Animals^29^ and approved under protocol no. 012/2015 by the Ethics Committee on Animal Use (EUA-UNP) under protocol no. 012/2015.

### Experimental design

After the isolation of the molecule of interest, experiments were performed to analyse the purification steps and to determine or estimate biochemical characteristics, such as molecular mass, amino acid sequence, alignment, stability, specificity parameters, mechanism of action, and isoelectric point. We also evaluated the purified inhibitor in an experimental model of obesity, observing its effect in plasmatic CCK and leptin in Wistar rats with obesity induced by a high-glycaemic index and glycaemic load diet.

### *Purification of the trypsin inhibitor from T. indica* L

This research followed an adapted protocol to obtain TTI^12^ and continued the purification according to the physical–chemical characteristics of the studied molecule. Peeled tamarind seeds had their cotyledons crushed in a refrigerated grinder (6 °C) until a fine-grained flour was obtained. This flour was solubilised in 50 mM Tris-HCl buffer, pH 7.5 at the ratio of 1:10 (w/v) under constant stirring for 3 h at 22 °C. After stirring, this homogenate was centrifuged at 12,000 rpm for 30 min at 4 °C. The precipitate from this centrifugation was discarded and the supernatant denominated the crude extract (CE).

The protein fractionation of the CE was performed by ammonium sulphate precipitation with the following saturation ranges: 0–30% (F1), 30–60% (F2), and 60–90% (F3). After each stage of precipitation, each saturation range was centrifuged for 30 min at 10,000 rpm, 4 °C. Each precipitate was resuspended in 50 mM Tris-HCl buffer, pH 7.5 and dialysed against distilled water for 20 h and against the same extraction buffer for further 20 h on a 12 kDa cut off pore membrane.

The fraction with the highest anti-trypsin activity, which was F2 (30–60%), was applied to a Trypsin-Sepharose affinity column (CNBr-activated Sepharose™ 4B, GE Healthcare, Little Chalfont, UK), pre-equilibrated with Tris-HCl (50 mM), pH 7.5. Non-retained proteins in the column were eluted with the same extraction buffer and the proteins retained in the matrix were eluted with HCl (5 mM) and collected in 5 ml aliquots. This eluate was dialysed against the extraction buffer and lyophilised, and TTI was obtained.

This set of trypsin inhibitors was applied and analysed by reversed-phase HPLC on a Shimadzu LC-10 A Liquid Chromatography consisting of a binary solvent pumping system (LC-10ADvp), UV-Vis spectrophotometric detector (SPD-10 A VP), Rheodyne injector and workstation with system control application (SCL-10Avp system controller configured with Lab solutions-LC solution Shimadzu software).

First, TTI was analysed in an analytical Shimadzu C18 (octadecylsilane) column (4.6 mm × 250 mm, 5 μm, 300 Å), with the following conditions: solvent A (0.1% of trifluoroacetic acid – 0.1%TFA/analytical grade water) and solvent B (60% of Acetonitrile – 60%ACN/0.1% TFA/analytical grade water), with linear gradient of 5 to 95% solvent for 30 min with percentage variation of ACN in solvent B of 3% min^−1^; flow rate of 1 ml min^−1^ and monitored by UV detection at 220 nm for peptide detection. From the analysis, the ideal percentage of solvent B for TTI was between 30 and 60% in 10 min with a percentage variation of ACN in solvent B of 3% B min^−1^. The purification was performed by injecting 150 μl of TTI at 2 mg mL^−1^ in a reverse-phase chromatography with a Vydac preparative C18 column (2.2 cm × 25 cm, 5 μm, 300 Å) with solvent A and solvent B at a flow rate of 9 ml min^−1^, and UV detection at 220 nm. Major protein components were manually collected.

The protein fraction obtained under the conditions described earlier, pure TTI (pTTI), was again subjected to a reverse-phase chromatography to amplify the separation resolution. 1 mg aliquot of pTTI was solubilised in 1 ml of analytical grade water, and applied onto a reverse-phase HPLC analytical column (Vydac C18, 4.6 × 250 mm, 5 μm, 300 Å). Solvent A (0.1% TFA/analytical grade water) and solvent B (60%ACN/0.1%/TFA analytical grade water) were used, with gradient from 5 to 65% B in 55 min (1.09% B min^−1^), and 65 to 95% B in 5 min (6%B min^−1^), with a flow rate of 1 ml min^−1^ and total time of 70 min. Elution of the protein content was monitored by UV detection at 216 and 280 nm. The protein samples were manually collected.

### Protein quantification and anti-trypsin activity

Protein quantification was done by the Bradford method using bovine serum albumin (BSA) as the standard^30^. Anti-trypsin activity was assessed according to Kakade et al. methodology^31^ using N-benzoyl-D,L-arginine-4-nitroanilide (BApNA) as the reaction substrate, 0.3 mg ml^−1^ of trypsin, determining the amount according to the titration curve, with amounts of inhibitor according to the test design. The negative control was a sample without addition of BApNA during the reaction. The assays were performed in triplicate.

### Fraction analysis from each purification step

The purification steps were analysed using 30 μg of protein from each step: CE, F2 (30–60%), TTI and pTTI, starting from 40 g of tamarind seed flour. Thus, volumes obtained in each step, the total amount of proteins, inhibitory units (IU), specific activity (IU.mg^−1^), degree of purification, and recovery were compared.

The inhibition unit (IU) was established by the difference between the total enzymatic activity of trypsin and the activity of the enzyme with CE, F2, TTI, and pTTI at absorbance from 0.01 to 410 nm. Thus, 1 IU was defined as the amount of inhibitor that decreased the absorbance by 0.01 optical density at the trypsin assay conditions^32^^,^^33^.

The results obtained with the CE were used as parameters for the determination of F2, TTI, and pTTI, with recovery 1 assigned to 100%. The purification, in number of times, was given by the ratio between the specific activities of each stage with the specific activity of the CE. The calculation was performed based on the ratio of the total protein quantity of each step to the total amount of proteins from the CE.

#### MALDI-TOF/MS

To analyse the level of purification and the molecular mass of each pTTI fraction, the pTTI fractions were applied in Bruker UltrafleXtreme III (Bruker Daltonics, Billerica, MA) spectrometer with MALDI ionisation source and TOF operation was conducted in the linear mode and molecular mass range from 2 to 23 kDa. Samples were prepared by mixing the fractions of pTTI and α-cyano-4-hydroxycinnamic acid matrix (α-CHCA) in the ratio 3:1 (v/v). The matrix was prepared in 50% ACN and 0.1% TFA. Data were acquired using Flex control and processed by FlexAnalysis (Bruker Daltonics, Germany) software version 3.4.

#### ESI-MS

To determine the accurate molecular mass of each pTTI fraction, mass spectrometry with electrospray ionisation source (ESI-MS) was used in the MicrOTOF-Q II (Bremen, Bruker Daltonics, Germany) spectrometer. pTTI fractions were solubilised in 200 µL of solvent A (1:1 H_2_O:Acetonitrile +0.1% Formic Acid) by direct infusion at the source. Data were processed by Compass DataAnalysis (Bruker Daltonics, Bremen, Germany) software version 4.3.110.

### Reduction and alkylation

The pTTI fractions were subjected to reduction by dithiothreitol (DTT, 50 mM) in ammonium bicarbonate solution (50 mM, pH 8.0) in water bath at 70 °C for 1 h. Then, for the alkylation of the thiol groups, 100 µl of iodoacetamide (IAM, 25 mM) was added to this solution to a concentration of 9.4 mg.mL^−1^. The reaction was kept at 37 °C for 1 h. The alkylation reaction was stopped by the addition of 5 μl of 50 mM of DTT. The pTTI fractions were then centrifuged at 13 400 rpm, for 5 min at 4 °C.

The reduced and alkylated pTTI fractions were subjected to chromatographic analysis. The procedure was performed by reversed-phase HPLC in an analytical column (Vydac C18, 4.6 mm ×250 mm, 5 μm, 300 Å). A gradient was used in which the first 15 min was 5% B to desalt, followed by a linear gradient of 5 to 65% B in 60 min (1%B.min^−1^); 65% to 95% B in 5 min (6% B.min^−1^), flow 1 ml.min^−1^, finishing the run with total time of 80 min. The reduced and alkylated pTTI fractions were manually collected, lyophilised, and solubilised in 200 μL of analytical grade water.

Aliquots of 1 μl of each reduced and alkylated pTTI fraction were analysed by MALDI-TOF and ESI-MS, as detailed above. In order to estimate the number of alkylated cysteines, a formula was applied using the molecular mass difference of the protein in its native form and its reduced and alkylated form, divided by the molecular mass of the carbamidomethyl group, which has 57.1 Da: N°Cys_alq_ = (M_alq_ − M_nat_)/57.1, in which: N°Cys_alq_ is the number of alkylated cysteines, M_alq_ is the molecular mass of the reduced and alkylated protein, M_nat_ is the molecular mass of the native protein and 57.1 is the molecular mass in Da of the carbamidomethyl group.

### Partial sequencing

From reduced and alkylated samples the fractions were solubilised in 10 μL of analytical grade water, 1 μL was combined with the 1,5-diaminonaphthalene (DAN) matrix at 6:1 (v/v). The matrix was prepared in 50% ACN and 0.1% TFA. After crystallisation, the analysis was done in an UltrafleXtreme spectrometer in the MALDI-TOF ionisation source by in-source decay (ISD) method. Manual peptide sequencing was performed using the Flex Analysis software (version 3.4.) to assign the -*c* and -*z* series.

### Multiple alignment

The obtained sequence was analysed and compared with sequences described in the NCBI (National Center for Biotechnology Information) non-redundant protein database, using BLASTp (Basic Local Alignment Search Tool − protein), with the multiple sequence alignment with sequences above 35% of identity in the database. The obtained alignment was processed using BioEdit Sequence Alignment Editor software, version 7.2.6.1^34^.

### Stability to denaturing agents

The methodology proposed by Gomes et al.^35^ was used to analyse the pTTI profile against pH extremes and temperature variation. For pH testing, 12 µg of pTTI was dialysed for 16 h, against the following buffers: glycine, pH 2, and pH 3; sodium phosphate, pH 6, and pH 8; glycine, pH 11, and pH 12 with a solute concentration of 100 mM. Subsequently, they were incubated for 30 min at 37 °C in the quenched buffers and dialysed over 4 h in 50 mM of Tris-HCl, pH 7.5. Then, the anti-trypsin inhibition assay^31^ was conducted. To ascertain the inhibitor stability at different temperatures, 12 µg of pTTI was incubated for 30 min at temperatures of 40, 60, 80, and 100 °C in water bath. The sample was cooled to 4 °C and then the anti-trypsin inhibition assay was performed^31^.

### Ki and IC50 against trypsin

Increasing amounts of pTTI (0.02, 1, 2, 3, 5, 6, 8 10 µg) were used in the anti-trypsin activity assay as previously mentioned^35^. The per cent of inhibition of each trypsin concentration was used for curve construction and the IC_50_ was estimated by probit regression, with the pTTI concentrations transformed to log10 base and the response as the per cent of inhibition. Data were processed using IBM SPSS Statistic 20 software.

*K*i was estimated by the intersection point between two lines, representing the two concentrations of BApNA (1.25 mM and 0.625 mM), according to the Dixon; Webb (1979) model^36^. The rate of enzymatic reaction (v) was expressed as the OD reaction product at 405 nm as a function of the reaction time (h) and volume (mL) as follows: *v* = OD h^−1^ mL^−1^.

### Isoelectric point

The pI estimation was performed as described by O’Farrell (1975)^37^, using 16 μg of pTTI. An immobilised gradient strip of pH 3 to 10 and 12% SDS-PAGE were used, as described by Laemmli (1970)^38^. The molecular mass marker ECLTM RainbowTM Marker – Full Ranger (AmershamTM) (12,000–225,000 Da) was used. The gel was revealed by Coomassie Blue staining. The pI estimation was performed by gel measurement, according to the scale size of the pH tape.

### Experimental model of obesity

Obese animals were randomly divided into two groups with five animals each: (1) Control Group, without treatment (C) (*n* = 5) and (2) Treatment group, with animals receiving pTTI (T) (*n* = 5). The animals were submitted to an adaptation period of five days, followed by ten days of treatment. Both groups received a high glycaemic index (77.6) and load (38.8) experimental diet for 15 days (during adaptation and treatment period), composed of Labina^®^, condensed milk, and refined sugar (4.5: 4.5: 1) (unpublished results).

During the 10 days of experiment and before treatments, the animals were daily fasted for 6 h. The control group received 1 ml of water per gavage/day and the Treatment group 1 ml of pTTI at a dose of 730 μg/Kg per gavage/day. This dosage was defined as a result of the pTTI IC_50_, following Carvalho et al.^12^ dosing recommendations, of 25 mg/Kg of TTI, also based on its IC_50_. On day 11, the animals were fasted for 8 h and anesthetised (250 mg tiletamine hydrochloride and 250 mg zolazepam hydrochloride). The animals’ blood was collected from the hepatic portal vein and animals were then euthanised. The collected blood was stored in tubes with EDTA and centrifuged at 3000 rpm for 10 min.

The dosages of CCK and leptin were performed by enzyme-linked immunosorbent assay (ELISA), as determined by the Enzyme Immunoassay Kit, Cholecystokinin (CCK)^26–33^ (Non-Sulphated) −(Phoenix Pharmaceuticals Inc., Burlingame, CA, USA) and Rat Leptin ELISA protocol (Millipore^®^-EZRL-83 K, Billerica, MA, USA).

### Statistical analysis

All data were obtained from at least three independent experiments and expressed as mean and standard deviation (SD), except when expressly indicated otherwise. The unpaired *T*-test was performed using the GraphPad Prism 6.0 software.

## Results

### *Purification of the trypsin inhibitor from T. indica* L

**Figure 1.**
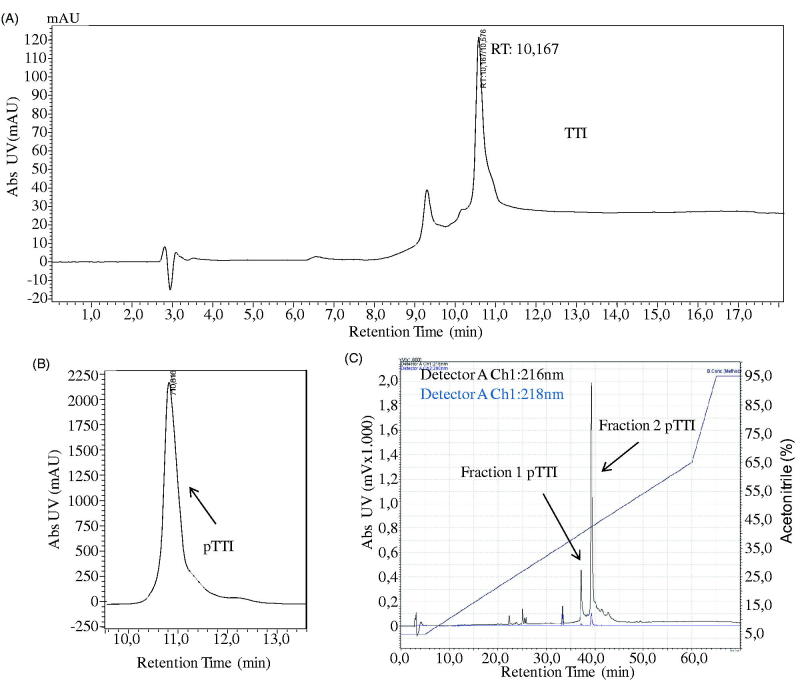
Chromatographic profile of TTI, pTTI, and its fractions in reversed-phase high-efficiency liquid chromatography. (A) Chromatographic profile of the partially purified inhibitor (TTI) in analytical C_18_ column. (B) Chromatographic profile of pTTI in preparative C_18_ column. (C) Chromatographic profile of fractions 1 and 2 by method refinement of pTTI in analytical C_18_ Vydac column. TTI: Trypsin inhibitor partially purified from tamarind seeds. pTTI: purified tamarind trypsin inhibitor from *T. indica* L.

The analytical C18 chromatogram demonstrates the protein profile of the purified inhibitor (Figure 1(A)). The protein peak corresponding to pTTI can be observed in Figure 1(B), with a retention time (RT) of 10 min and elution with 37.4% of ACN.pTTI was again injected into a reverse-phase analytical column (Vydac C18, 4.6 × 250 mm, 5 μm, 300 Å), increasing resolution. Thus, a total of 140 μg/500 μl was injected and two major protein peaks were observed (Figure 1(C)). These were denominated fractions of pTTI, fraction 1 (Fr1), and Fraction 2 (Fr2). Fr1 presented with an RT of 37 min and Fr2 of 39 min. The percentage of solvent B was 45.5% and 47.8%, respectively.

### Fraction analysis from each purification step

The purification steps were evaluated and are presented in Table 1. A reduction of the total amount of soluble proteins can be observed between the steps, concomitantly increasing the specific activity (IU.mg^−1^) of pTTI. In addition, it is possible to observe a recovery of approximately 2% of the total protein contained in the crude extract (CE).

**Table 1.** Purification steps of pTTI from *T. indica* L. seeds.

Steps	Volume (ml)	Total Protein (mg)	Total Protein in assay (mg)	Concentration (mg/ml)	Inhibition units (IU)	Specific activity (IU.mg^−1^)	Purification (x)	Recuperation (%)
Crude extract	354	644	0.03	1.82	27.5	915	1	100
F2 (30–60%)	165	292	0.03	1.77	23.8	794	0.87	45.3
TTI	627	53.3	0.015	0.085	25.8	1720	1.88	8.27
pTTI	4.6	12.8	0.012	2.8	26.4	2196	2.40	1.99

F2(30–60%): Fraction 2 with ammonium sulphate saturation of 30–60%; TTI: trypsin inhibitor isolated from *T. indica* L; pTTI: purified tamarind trypsin inhibitor from *T. indica* L.

#### MALDI-TOF

**Figure 2.**
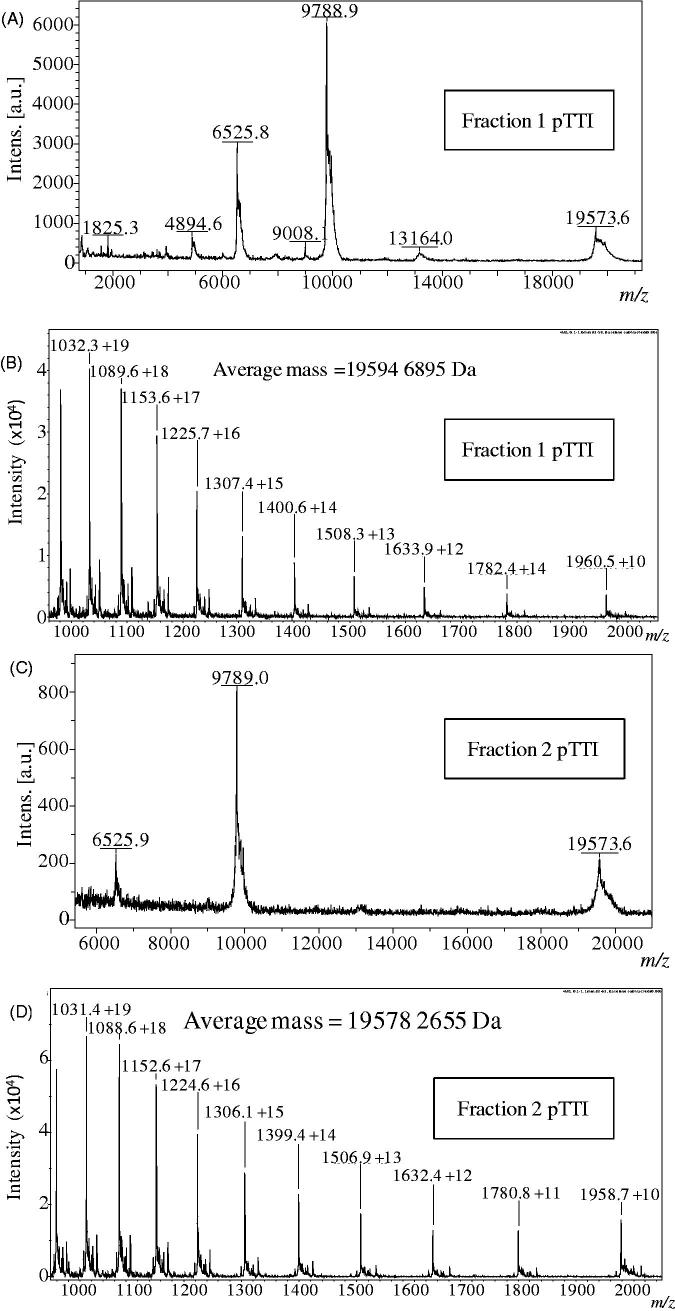
MALDI-TOF and ESI mass spectra of fractions 1 and 2. Mass spectra in MALDI-TOF, linear acquisition mode of (A) Fraction 1 and (B) Fraction 2. ESI MS mass spectrum of (C) Fraction 1 and (D) Fraction 2. pTTI: Purified tamarind trypsin Inhibitor from *T. indica* L.

The MALDI-TOF average molecular mass spectrum of each fraction of pTTI is shown in Figures 2(A,C). Figure 2(A) shows Fr1, which presented different masses that possibly represent three charge states of the same molecule, [M + H]^+^ = 19,573 Da; [M + 2H]^+^ = 9788 Da and [M + 3H]^+^ = 6525 Da. Fr2 when analysed under the same conditions as Fr1, also presented different masses that may represent three charge states of the same molecule, [M + H]^+^ = 19,573 Da; [M + 2H]^+^ = 9789 Da and [M + 3H]^+^ = 6528 Da (Figure 2(C)). Besides these ions, we observed in the spectra of the two fractions, two ions with 13,164 Da and 1315 Da. However, these molecular components could not be unequivocally determined.

#### ESI-MS

The average molecular masses analysed in MicroTOF-Q II spectrometer, with ESI ionisation are shown in Figure 2(B,D). The molecular mass of Fr1 was [M + 14H]^+^ = 19,594.689 Da (Figure 2(B)) and Fr2 was [M + 13H]^+^ = 19,578.266 Da (Figure 2(D)). The molecular mass difference between the fractions is 16.423 Da.

### Reduction and alkylation

**Figure 3.**
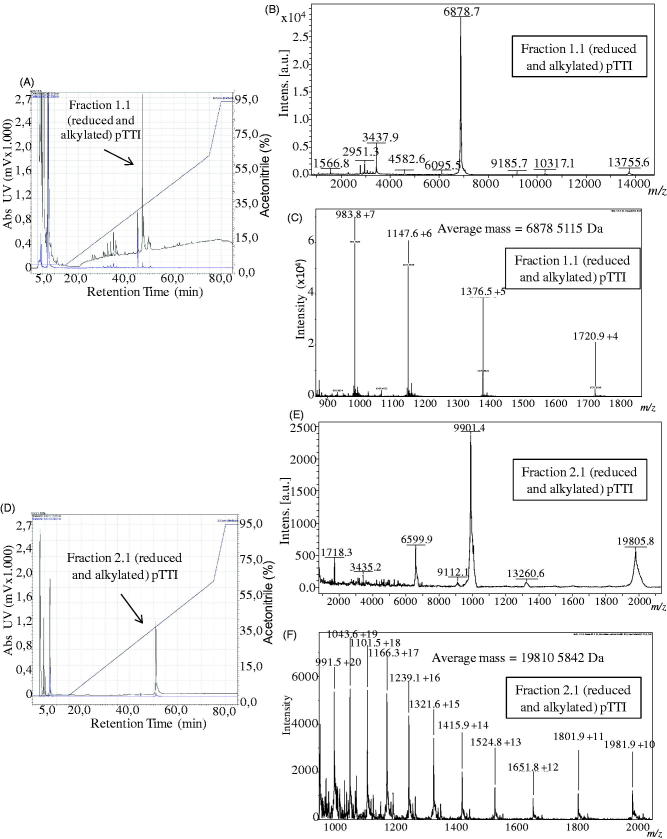
Chromatograms, mass spectra with MALDI-TOF ionisation source and ESI spectra in micrOTOF-Q II of reduced and alkylated fractions 1 and 2 (Fr1.1 and Fr2.1). (A) Chromatogram of reduced and alkylated Fr1. (B) MALDI-TOF mass spectrum of Fr1.1, with three major charge states. (C) ESI mass spectrum of Fr1.1, showing its protonated molecular mass. (D) Chromatogram of reduced and alkylated Fr2. (E) MALDI-TOF mass spectrum of Fr2.1, with three charge states. (F) ESI mass spectrum of Fr2.1, showing its protonated molecular mass. pTTI: Purified tamarind trypsin Inhibitor from *T. indica* L.

The fractions Fr1 and Fr2 when reduced and alkylated showed a protein peak of higher absorbance with RT of 46 min and 51 min and concentration of solvent B of 36.9% and 41.1%, and were denominated Fr1.1 and Fr2.1, respectively (Figure 3(A,D)).

**Figure 4.**
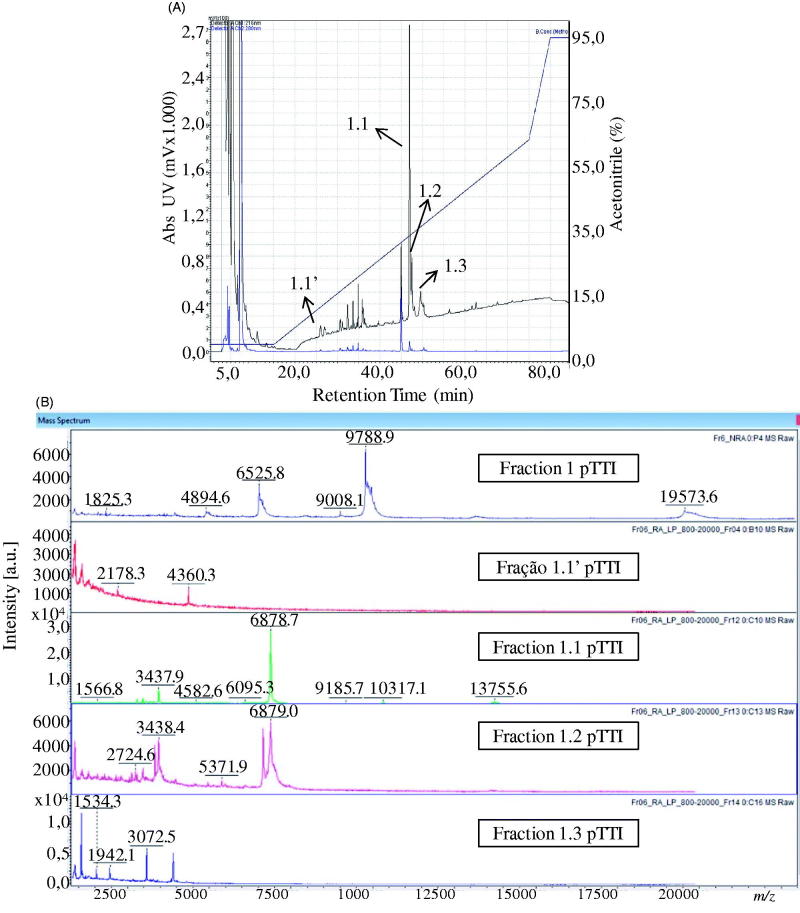
Chromatogram and spectra in MALDI-TOF fractions obtained from Fr1 after reduction and alkylation. (A) Chromatogram of Fr1 after reduction and alkylation, with designation of the purified fractions. (B) Spectra acquired in MALDI-TOF from purified fractions of the reduced and alkylated Fr1 with respective chromatogram designation.

The fractions Fr1.1 and Fr2.1 were analysed by MALDI-TOF to investigate the reduction and alkylation reaction. Fr1.1 presented two predominant ions with *m/z* = 6878 Da and *m/z* = 3437 Da (Figure 3(B)). When compared to the Fr1 (native) spectrum, it was observed that there was no ion with mass near 19.8 kDa, which would represent the reduced and alkylated molecule (Figure 4). Thus, it was not possible to infer the charge state of the two ions, and therefore to estimate the amount of cysteines in this molecule, which leads us to the hypothesis that the ion with 19,594.690 Da observed in the native fraction may be a trimer of the ion with 6525 Da, and this, in turn, is a fragment of Fr2.

The fraction Fr2.1, presented ions whose masses *m/z* were 19,805 Da, 9901 Da, and 6600 Da, which can represent three charge states, mono, double, and triple-charged, respectively (Figure 3(E)). In addition, they corroborate with the expected masses for reduced and alkylated Fr2 and show the presence of four cysteines, which favour the presence of two disulphide bonds.

The molecular masses analysed in MicroTOF-Q II spectrometer with ESI ionisation source of Fr1.1 and Fr2.1 are presented in Figures 3(C,F). The molecular mass of Fr1.1 was [M + H]^+^ = 6878.511 Da and of Fr2.1 was [M + H]^+^ = 19,810.584 Da.

### Partial sequencing

**Figure 5.**
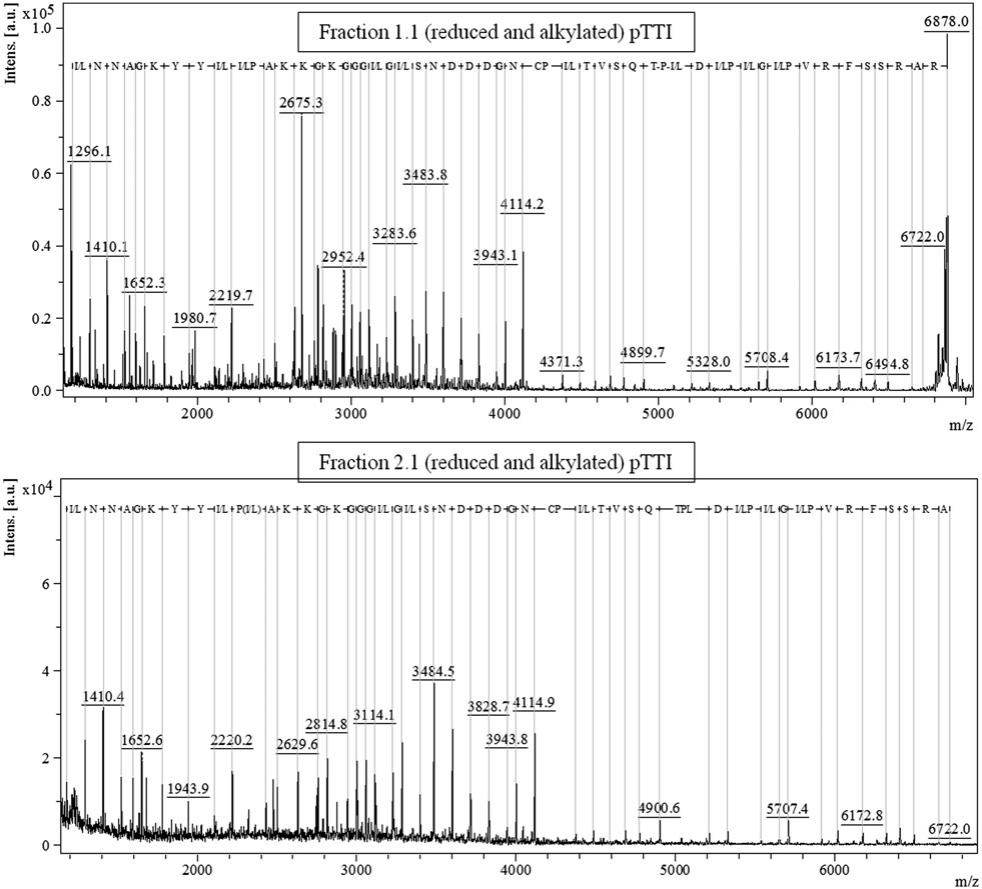
Sequencing by in-source decay MALDI-TOF of reduced and alkylated pTTI fractions, exhibiting the amino acid residue sequence above the 1000 *m/z* region. (A) Fr1.1 spectrum. (B) Fr2.1 spectrum. Sequences obtained comprised 53 amino acid residues, identical for both fractions. pTTI: purified tamarind trypsin inhibitor from *T. indica* L.

Fractions Fr1.1 and Fr2.1, those reduced and alkylated, were partially sequenced by in-source decay in MALDI-TOF MS, using DAN as matrix. The spectrum with the primary partial sequence of Fr1.1 is shown in Figure 5(A), the obtained signal had a molecular mass of [M + H]^+^ = 6878 Da, and the spectrum of Fr 2.1, in Figure 5(B), had a molecular weight of [M + H]^+^ = 6722 Da.

The possibility that Fr1 is a fragment of Fr2, becomes more consistent when a parental ion of [M + H]^+^ = 6878 Da is observed in the partial sequence of Fr1.1, which resulted in a primary sequence with 54 amino acid residues (Figure 5(A)).

In Fr2.2, also partially sequenced, in the spectrum of this sequencing no parental ion is observed, evidencing that the molecular mass of Fr2.1 is larger than the fragment analysed, whose sequence was obtained from the ion with [M + H]^+^ = 6722 Da (Figure 5(B)).

The sequence obtained contains 53 amino acid residues and is the same as the sequence obtained for Fr1.1, further corroborating the hypothesis that this is a fragment of Fr2.1. Moreover, these results are also evidenced by the ESI spectra, which shows an accurate mass of [M + 5H]^+^ = 6878.511 Da (Fr1.1) and [M + H]^+^ = 19,810.584 Da (Fr2.1) (Figure 2(C,D)).

The partial sequence obtained presented the sum of the average molecular mass of the resulting amino acid residues of 5582.345 Da, representing about 81% and 84% coverage of the molecular mass obtained for Fr1.1 and Fr2.1, respectively. The sequence obtained is composed of 54 and 53 amino acid residues as following:

Fr1.1:

(I/L)NNAGKYY(I/L)(I/L)PAKKGKGGG(I/L)G(I/L)SNDDDGNCP(I/L)-TVSQTP(I/L)D(I/L)P(I/L)G(I/L)PVRFSSRAR.

Fr2.1:

(I/L)NNAGKYY(I/L)(I/L)PAKKGKGGG(I/L)G(I/L)SNDDDGNCP(I/L)TVSQTP(I/L)D(I/L)P(I/L)G(I/L)PVRFSSRA.

Because, the molecular masses of leucine and isoleucine are identical, with 113.160 Da, it is not possible to distinguish between these residues.

### Multiple alignment

The search in the database of the 54 amino acid residues of pTTI resulted in several possible sequences of alignment. Alignment is shown in Figure 6. The primary sequence of pTTI lies between residues 40 and 100 when compared to other aligned sequences. The highest percentages of identity were with the sequences of kunitz family trypsin inhibitor from tamarind, the first three sequences of Figure 6, with 91, 91, and 89% of identity. The other sequences showed between 35 and 56% of identity. The smaller e-value was for the tamarind itself, *Copaifera Langsdorffii* (popularly known as Copaíba) and *Glycine Max* (soybean).

Figure 6.Multiple alignment of the primary partial sequence of pTTI with other sequences deposited in the NCBI. pTTI: purified tamarind trypsin inhibitor from *T. indica* L.

**Figure:**
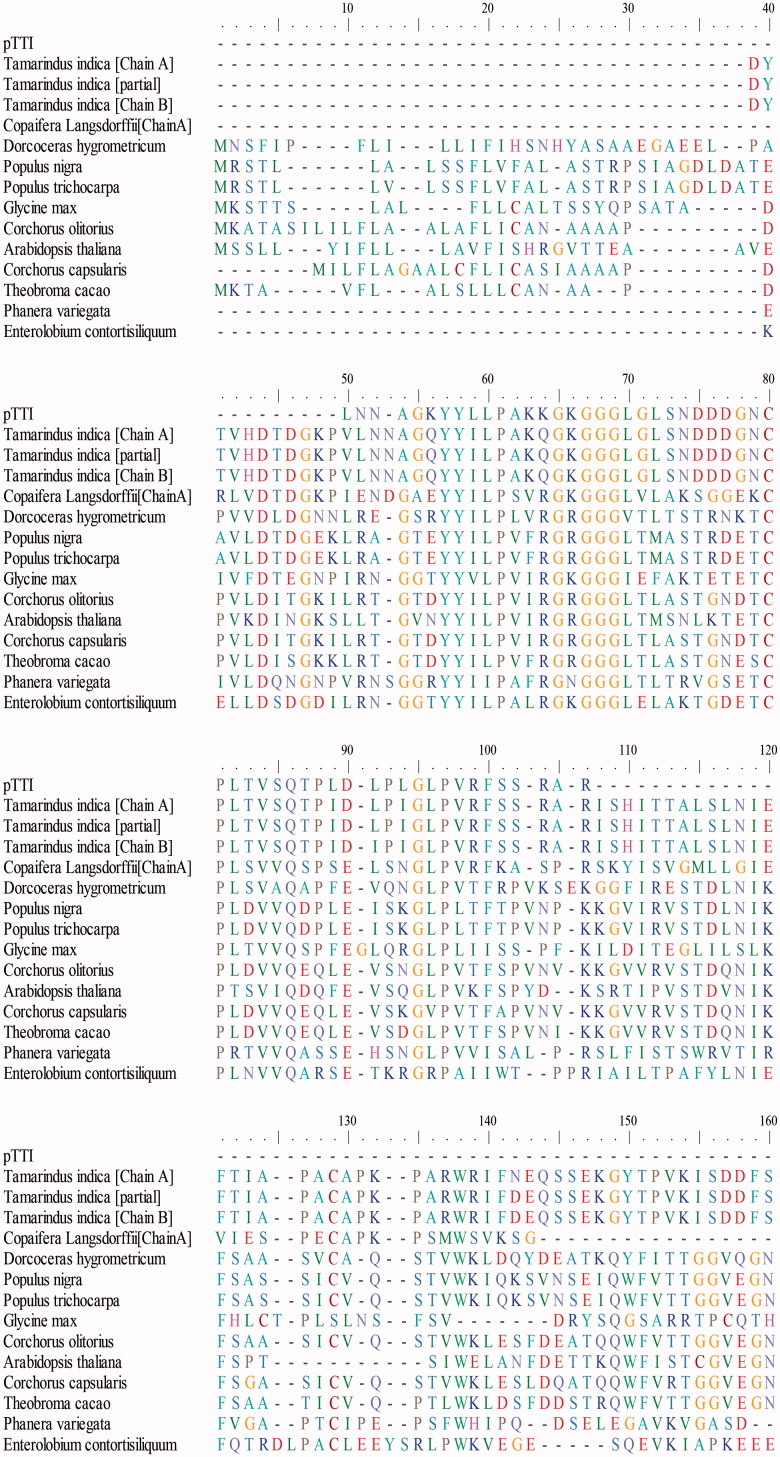

**Figure:**
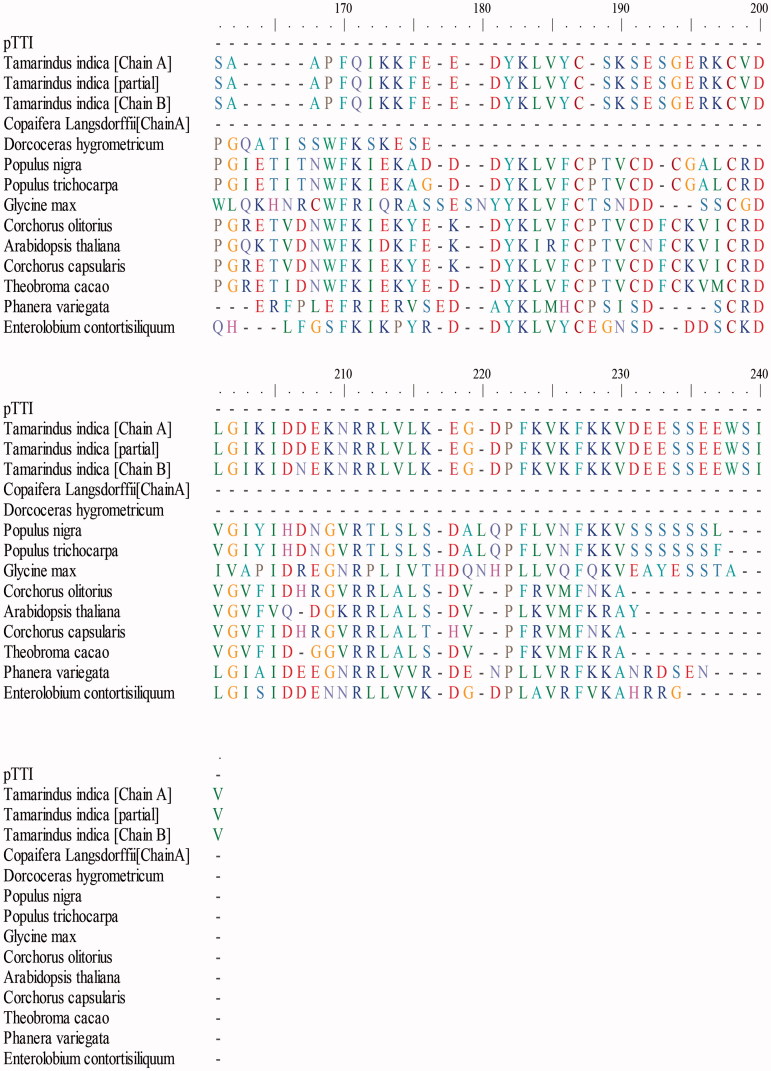

### Stability to denaturing agents

**Figure 7.**
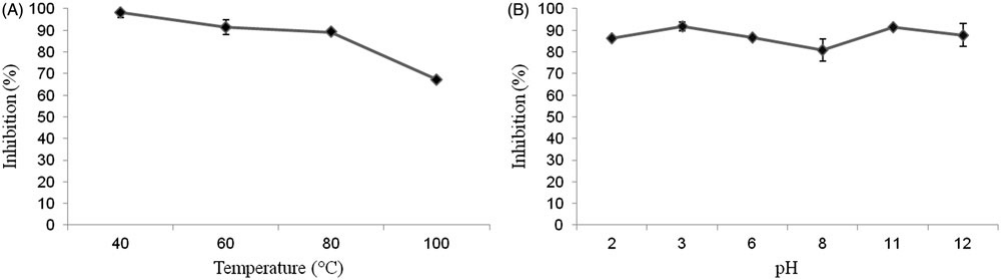
(A) Stability of pTTI at different temperatures. (B) Stability of pTTI at different pH. pTTI: purified tamarind trypsin inhibitor from *T. indica* L.

Thermal and pH variation stabilities were assessed by intracellular *in vitro* activity of pTTI. In the heating test, pTTI maintained its antitrypsin activity, except at 100 °C, still maintaining about 70% of its activity compared to that presented at the temperature of 40 °C. Activities at temperatures 60 and 80 °C were maintained, with a small reduction, as shown in Figure 7(A). In the pH assay, stability was also seen, with a small reduction of the antitrypsin activity in the range of pH 6 to pH 8, which may be due to the pI of this inhibitor (Figure 7(B)).

### Ki and IC50 against trypsin

**Figure 8.**
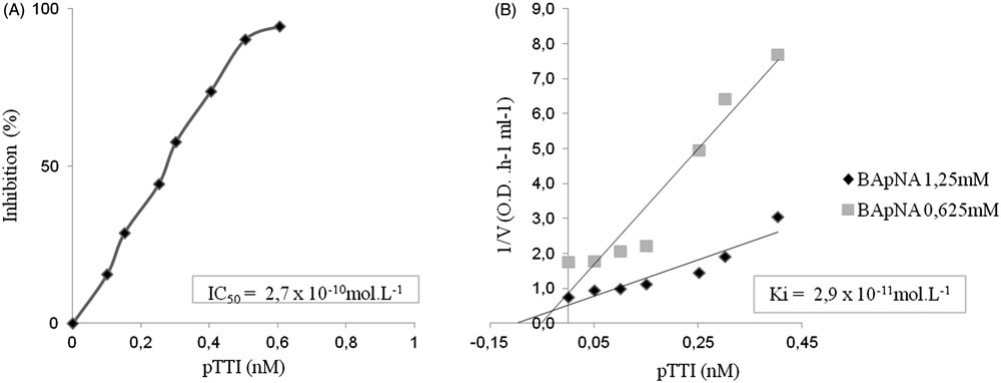
Estimation of pTTI specificity parameters. (A) Trypsin inhibition curve using increasing amounts of pTTI. (B) Estimation of inhibition kinetics and pTTI trypsin inhibition constant. pTTI: purified tamarind trypsin inhibitor from *T. indica* L.

The IC50 resulted in 2.7 × 10^−10 ^mol L^−1^ pTTI to inhibit trypsin activity by 50% (Figure 8(A)). The *K*_i_ value was of 2.9 × 10^−11 ^mol L^−1^. As shown in Figure 8(B), the inhibition assays indicated that pTTI acts as a competitive inhibitor.

### Isoelectric point

**Figure 9.**
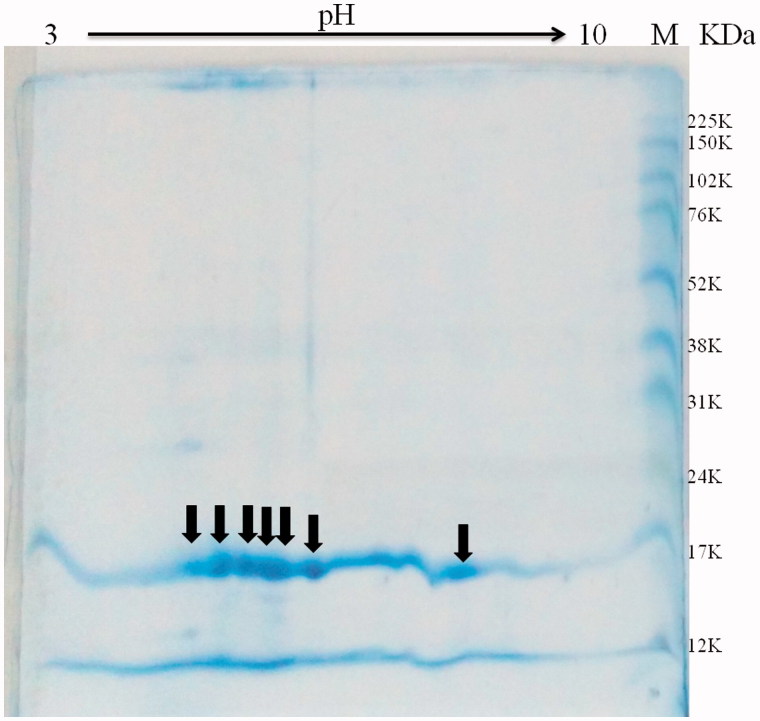
Two-dimensional electrophoresis of pTTI with gradient strips of pH 3–10. Arrows indicate the spots estimated between pH 5 and 6, in addition to another with a pH of approximately 8. pTTI: purified tamarind trypsin inhibitor from *T. indica* L.

The pI was estimated from the analysis of two-dimensional electrophoresis gel. 16 µg pTTI was applied to the two-dimensional gel. It was possible to observe some spots near pH 5 and 6, 18 kDa, and another spot near pH 8 (Figure 9).

### Experimental model of obesity

Plasma concentrations of CCK and leptin are shown in Table 2. For CCK concentrations, a slight increase in the pTTI-treated group can be observed (not significant). We observed that 60% of the obese animals receiving pTTI (*n* = 03) had very low leptin concentrations, under the minimum detection concentration (< 0.1 ng/mL). Thus, the only two obese animals with detectable plasma leptin presented 0.28 ng/mL of this hormone, also very low, however, detectable by the test. This made comparing mean plasma leptin concentrations between studied groups unfeasible. In the untreated group, 100% animals had values above the detectable level (>0.1 ng mL^−1^) (Table 2).

**Table 2.** Plasma concentrations of CCK and leptin from obese Wistar rats treated with pTTI for 10 days.

Groups	Plasmatic CCK (ng/ml) Mean (SD)	Plasmatic leptin (ng/ml) Mean (SD)
Control (without treatment)	2.03 (0.49)^a^	0.50 (0.14)
Treatment with pTTI	2.51 (0.27)^a^	0.28^b^

The groups received a high glycaemic (77.6) and load (38.8) index diet. There was no statistical difference between groups for CCK, *p* > .05, unpaired *T* test. pTTI: Purified tamarind trypsin inhibitor from *T. indica* L.

a There was no significant difference between groups.

b 60% of animals in this group had plasma leptin values below the detection concentration (0.1 ng/mL) and the standard deviation was not calculated.

## Discussion

Characterising a peptidase inhibitor is fundamental for its classification and knowledge of its properties, important for further investigation of therapeutic features. Several peptidase inhibitors have been characterised and studied in human diseases, such as acquired immune deficiency syndrome (AIDS), Alzheimer’s, osteoporosis, cardiovascular diseases, rheumatoid arthritis, cancer, obesity, and others^12^^,^^39–42^.

Once there are reports of anti-obesity and anti-inflammatory effects of crude inhibitors isolated from the aqueous extracts of the *T. indica* L. seed in eutrophic and obese Wistar rats^11^^,^^12^, we decided to observe the effects of the purified trypsin inhibitor (pTTI). In addition, the chemical and structural analyses of pTTI were performed to identify this compound and characterise its inhibition mechanism.

In the pTTI purification process, the specific activity data, in which the purified inhibitor has a higher IU mg^−1^ protein compared to the previous steps, demonstrated the increase in the inhibition potential of this protein as the purification process occurs, and Fr1.1 and 2.1 were not tested independently.

The recovery is also an item to be highlighted, which was approximately 2% higher than the starting CE. The yield of the purification process is an important data in the study with these inhibitors with bioactive potential since they involve several steps. Well delimited, high reproducible steps are crucial and must be carried out to achieve the highest possible yield^43^.

Thus, pTTI was purified and the molecular masses, measured by the ESI ionisation technique for Fr1 and Fr2 in their native form, were within the characteristic range for trypsin inhibitors of the Kunitz family, between 18 and 22 kDa^42^. A trypsin inhibitor has already been purified from tamarind seeds, but the molecular mass analysed by MALDI-TOF was 21,420 Da, different from the values obtained in this study^26^. In addition, we observed that pTTI presented two protein fractions, with very close molecular masses and identical amino acid sequences. After reduction and alkylation, the obtained molecular masses were smaller than expected. Nevertheless, the results give further evidence that pTTI is a Kunitz-type inhibitor because it showed about four Cys residues and thus possibly two disulphide bonds^42^. Taken altogether, the results indicate that Fr1 is, possibly, a fragment of Fr2.

Molecular masses of purified pTTI (Fr1 and Fr2) obtained in their native form by the ESI ionisation technique were 19.594 and 19.578 kDa which fall in the range of 18 to 22 kDa, of the Kunitz family trypsin inhibitors^42^. In other study, a different trypsin inhibitor purified from tamarind seeds, also analysed by MALDI-TOF, exhibited a molecular mass of 21,420 Da^26^.

We observed that pTTI consisted in two protein fractions, with similar molecular masses and identical amino acid sequences. Besides that, molecular masses of these compounds submitted to reduction and alkylation were smaller than expected. These results indicated that Fr1 could be a fragment of Fr2.

As known, the reduction and alkylation reaction made the estimation of the number of Cys present in the Fr2.1 structure possible. The results shown in those assays give further evidence that pTTI is a Kunitz-type inhibitor with about four Cys, which are capable to form two disulphide bonds^42^.

The analysis by MALDI-TOF ISD mode for partial obtainment of the major protein peaks primary structure after reduction and alkylation reinforce that Fr1 and Fr2 are the same molecule, because their amino acid sequences were the same. In the ISD technique, cleavage of the N-Cα bond occurs, resulting in the -*c* and -*z* fragments. ISD has been commonly applied for protein sequencing since very small molecules have low *m/z* region and the analysis becomes incomprehensible due to matrix agglomerates, which makes peptide sequencing difficult^21^. Therefore, the Fr1 and Fr2 sequences of amino acid residues were possible to visualise only above 1000 *m/z* region of the MALDI TOF mass spectrometer.

In the multiple alignment using BLASTp, we found a tamarind trypsin inhibitor partially sequenced from cDNA. Comparing these sequences, none gaps, high identity, and low e-value were observed. Gaps represent deletions or insertions and the high identities were of 91% for the first two sequences and of 89% for the third one. Thus, the obtained sequence matches those already deposited. Alignment with the other sequences showed greater identity with plant and seed Kunitz trypsin inhibitors. Considering that in this study the purified fraction 1 is the fragment of fraction 2, we suggest that pTTI belongs to the Kunitz family of inhibitors.

Additionally, the kinetic study characterised the enzymatic mechanism of pTTI. The specificity parameters give important information about the inhibitor and the peptidase mechanism of action. pTTI *K*_i_ was of 2.9 × 10^−11 ^mol L^−1^, and Dixon plot^36^ indicated that it is a competitive inhibitor^44^. The trypsin inhibitor purified by Araújo et al.^26^ presented a *K*_i_ value of 1.7 × 10^−9 ^mol L^−1^, with noncompetitive kinetics, demonstrating that it is different from the inhibitor purified in this study, even though they originated from the same seed.

Comparing these two trypsin inhibitors, pTTI is a molecule that has a strong competitive interaction with trypsin and therefore is a candidate inhibitor for the pharmaceutical industry, since inhibitors with this characteristic are commonly noncompetitive^45^. Many drugs have competitive kinetics: statins are a classic example, reversibly competing with 3-hydroxy-3-methyl-glutaryl-coenzyme A (HMG-CoA) for the active site of the enzyme HMG-CoA reductase, inhibiting cholesterol synthesis^46^.

Concerning pTTI IC_50_ value of 2.7 × 10^−10 ^mol L^−1^, it is possible to compare it to other trypsin inhibitors. Some studies have shown IC_50_ values of purified trypsin inhibitors, such as EvTI with an IC_50_ of 2.2 × 10^−8 ^mol L^−1^ (32), and the *Entada scandens* seed trypsin inhibitor (ESTI) with 2.6 × 10^−8 ^mol L^−1^^47^. Thus, compared to other trypsin inhibitors, pTTI is more potent, which favours bioprospecting investigations.

Our results showed pTTI presented resistance to pH extremes, maintaining its anti-trypsin activity in both acid and alkaline media. A reduction in the percentage of inhibition is observed only between pH 6 and 8. This can be explained by pTTI pI, which determines that in this pH range a reduction of the electrostatic repulsion is expected: null loads lead to a reduction in electrostatic repulsion, leading to aggregation of pTTI, explaining the reduction of its anti-trypsin activity in this pH range^48^. pTTI maintained its anti-trypsin activity relatively stable until 80 °C, which characterises a heat-resistant protein. When at 100 °C there is a reduction of its activity, but 67% inhibition of trypsin is still maintained.

The thermo-resistance of a protein is a characteristic of fundamental importance since several biotechnological processes are carried out at high temperatures, such as the synthesis of a new drug, and therefore, the more the inhibitor’s thermo-stability, the more promising it is for a large-scale production^49^.

Two-dimensional electrophoresis of pTTI showed some spots with pI between pH 5 and 6, and one spot in the region of pH 8. These spots might indicate isoforms and can be numerous, what makes the process of identification a challenge^50^.

Functional reassessment of pTTI on CCK and leptin plasma concentrations in obese Wistar rats confirmed Costa et al.^25^ previous findings with the partially purified inhibitor. In the study of Costa et al.^25^, the inhibitor also reduced plasma leptin but no changes in plasma CCK were observed. Interestingly, considering the minimum detection level of leptin in this study, untreated obese animals had leptin concentrations at least five times higher than 60% of animals treated with pTTI. These findings corroborate studies in obese humans and rodents, showing that leptin concentrations in obesity can be five times higher than in eutrophy^51^^,^^52^.

Resistance to leptin with consequent hyperleptinaemia are metabolic conditions observed in obesity^53^. Lartigue et al.^3^ demonstrated the importance of the interaction of CCK with leptin and of reducing leptin resistance in obese animals to improve CCK satiety action.

TTI, partially purified in a previous study^25^, and pTTI, the purified molecule evaluated in this study, did not cause an increase in CCK. However, with the trypsin inhibitor still partially purified, a significant reduction in food consumption, without a decrease in weight gain, was observed in 10 days of experiment, for the obese animals^12^. Thus, the hypothesis that the action of TTI and pTTI are related to satiety in obesity is likely dependent on the action of these inhibitors on leptin and not on CCK, as seen for eutrophic animals^11^.

It is worth mentioning that the reduced sensitivity to CCK in obese animals is considered a factor for the continuity of this metabolic condition since even in physiological CCK doses administered to rats with obesity, there is no satietogenic effect as there is in eutrophic animals^54^. In diet-induced obesity in Sprague–Dawley rats, weight reduction was observed only in 23 days after exogenous CCK-8 was received^55^.

Possibly, in a longer experimental time, the CCK secretagogue isolated or purified from tamarind seeds could present better results regarding appetite control and, consequently, weight loss, considering that TTI decreased food consumption in animals with obesity^12^. Moreover, a longer period of treatment, raising or not CCK, by the reduction in CCK resistance promoted by hyperleptinaemia in animals with obesity could culminate in an additive effect on reduced food consumption^12^, and consequent reduced weight gain.

Obese animals with reduced leptin concentrations are more sensitive to endogenous leptin and CCK^3^^,^^56^, so a reduction in plasma leptin is a promising effect of pTTI, as part in the satiety signalling. CCK may also have its signalling effect potentiated by leptin, via the vagus nerve. It is known that high leptin concentrations cause a reduction in the sensitivity of the afferent vagal neurons to CCK^3^. Therefore, the reduction of leptin resistance seems to be a positive pathway for the satietyogenic effects of CCK in obese animals, favouring a better sensitivity to the effect of CCK.

Carvalho et al.^12^, in a study with obese animals with metabolic syndrome (MetS) receiving TTI (25 mg/Kg), demonstrated that this inhibitor reduced TNF-α plasma concentrations, independently of weight loss. It is known that TNF-α is an inflammatory cytokine synthesised mainly by the white adipose tissue, remarkably in individuals with obesity^57^. The results obtained extrapolated TTI action to those related to its anti-trypsin activities and the consequent CCK increase. This suggests that the purified molecule may exhibit an important anti-inflammatory effect and this will be further addressed in other studies.

Thus, the biochemical knowledge of this molecule and its effect on leptin in an experimental model of obesity provides essential and innovative information for a biomolecule of possible biotechnological application, once the knowledge of its amino acid sequence facilitates its biotechnological synthesis.

## References

[1] WHO Obesity and overweight. Geneva: The World Health Organization. 2016;1–5. Available from: http://www.who.int/mediacentre/factsheets/fs311/en/

[2] GBD Health effects of overweight and obesity in 195 countries over 25 years. N Engl J Med 2017;377:13–27. 28604169 10.1056/NEJMoa1614362 PMC5477817

[3] Lartigue G, Serre CB, Espero E, et al Leptin resistance in vagal afferent neurons inhibits cholecystokinin signaling and satiation in diet induced obese rats. PLoS One 2012;7:1–10. 10.1371/journal.pone.0032967 PMC3296757 22412960

[4] Niederau C, Meereis-Schwanke K, Klonowski-Stumpe H, et al CCK-resistance in Zucker obese versus lean rats. Regul Pept 1997;70:97–104. 9272621 10.1016/s0167-0115(97)00014-1

[5] Duca FA, Zhong L, Covasa M. Reduced CCK signaling in obese-prone rats fed a high fat diet. Horm Behav 2013;64:1–6. 24100196 10.1016/j.yhbeh.2013.09.004

[6] Rehfeld JF Clinical endocrinology and metabolism. Cholecystokinin. Best Pract Res Clin Endocrinol Metab 2004;18:569–86. 15533776 10.1016/j.beem.2004.07.002

[7] Park HK, Ahima RS. Physiology of leptin: energy homeostasis, neuroendocrine function and metabolism. Metabolism 2015;64:24–34. 25199978 10.1016/j.metabol.2014.08.004 PMC4267898

[8] He N-W, Zhao Y, Guo L, et al Antioxidant, antiproliferative, and pro-apoptotic activities of a saponin extract derived from the roots of Panax notoginseng (Burk.) F.H. Chen. J Med Food 2012;15:350–9. 22316295 10.1089/jmf.2011.1801 PMC3308717

[9] Komarnytsky S, Cook A, Raskin I. Potato protease inhibitors inhibit food intake and increase circulating cholecystokinin levels by a trypsin-dependent mechanism. Int J Obes 2011;35:236–43. 10.1038/ijo.2010.192 PMC3033477 20820171

[10] Nakajima S, Hira T, Tsubata M, et al Potato extract (Potein) suppresses food intake in rats through inhibition of luminal trypsin activity and direct stimulation of cholecystokinin secretion from enteroendocrine cells. J Agric Food Chem 2011;59:9491–6. 21809886 10.1021/jf200988f

[11] Ribeiro J, Serquiz A, Silva P, et al Trypsin inhibitor from tamarindus indica L. seeds reduces weight gain and food consumption and increases plasmatic cholecystokinin levels. Clinics 2015;70:136–43. 25789523 10.6061/clinics/2015(02)11 PMC4351314

[12] Carvalho FMC, Lima VCO, Costa IS, et al A trypsin inhibitor from tamarind reduces food intake and improves inflammatory status in rats with metabolic syndrome regardless of weight loss. Nutrients 2016;8:1–14. 10.3390/nu8100544 PMC5083972 27690087

[13] Chen W, Hira T, Nakajima S, et al Suppressive effect on food intake of a potato extract (Potein®) involving cholecystokinin release in rats. Biosci Biotechnol Biochem 2012;76:1104–9. 22790930 10.1271/bbb.110936

[14] Serquiz AC, Machado RJA, Serquiz RP, et al Supplementation with a new trypsin inhibitor from peanut is associated with reduced fasting glucose, weight control, and increased plasma CCK secretion in an animal model. J Enzyme Inhib Med Chem 2016;31:1261–9. 26928305 10.3109/14756366.2015.1103236

[15] Farady CJ, Craik CS. Mechanisms of macromolecular protease inhibitors. Chembiochem 2010;11:2341–6. 21053238 10.1002/cbic.201000442 PMC4150018

[16] Rawlings ND, Tolle DP, Barrett AJ. Evolutionary families of peptidase inhibitors. Biochem J 2004;378:705–16. 14705960 10.1042/BJ20031825 PMC1224039

[17] Souza DD, Brandão-Costa RMP, Albuquerque WWC, et al Partial purification and characterization of a trypsin inhibitor isolated from *Adenanthera pavonina* L. seeds. South African J Bot 2016;104:30–4.

[18] Ryan CA. Protease inhibitors in plants: genes for improving defenses against insects and pathogens. Annu Rev Phytopathol 1990;28:425–49.

[19] Fan S-G, Wu G-J. Characteristics of plant proteinase inhibitors and their applications in combating phytophagous insects. Bot Bull Acad Sin 2005;46:273–92.

[20] Oliveira AS, Pereira RA, Lima LM, et al Activity toward Bruchid Pest of a Kunitz-Type inhibitor from seeds of the Algaroba Tree (*Prosopis juliflora* D.C.). Pestic Biochem Physiol 2002;72:122–32.

[21] Köche T, Engström Å, Zubarev RA. Fragmentation of peptides in MALDI in-source decay mediated by hydrogen radicals. Anal Chem 2005;77:172–7. 15623293 10.1021/ac0489115

[22] Lanças FM. A cromatografia líquida moderna e a espectrometria de massas: finalmente “compatíveis”? Sci Chromatogr 2009;5:27–46.

[23] Fook JMSLL, Macedo LLP, Moura GEDD, et al A serine proteinase inhibitor isolated from *Tamarindus indica* seeds and its effects on the release of human neutrophil elastase. Life Sci 2005;76:2881–91. 15820500 10.1016/j.lfs.2004.10.053

[24] Pandey PK, Jamal F Bio-potency of a 21 kDa Kunitz-type trypsin inhibitor from Tamarindus indica seeds on the developmental physiology of *H. armigera*. Pestic Biochem Physiol 2014;116:94–102. 25454525 10.1016/j.pestbp.2014.10.001

[25] Costa IS. Efeito de proteínas bioativas isoladas do tamarindo secretagogas da CCK e seu sinergismo com leptina em ratos Wistar obesos. Universidade Federal Do Rio Grande Do Norte, Brazil; 2017.

[26] Araújo CL, Bezerra IWL, Oliveira AS, et al In vivo bioinsecticidal activity toward *Ceratitis capitata* (Fruit Fly) and *Callosobruchus maculatus* (Cowpea Weevil) and in vitro bioinsecticidal activity toward different orders of insect pests of a trypsin inhibitor purified from tamarind tree (*Tamarindus indica*) seeds. J Agric Food Chem 2005;53:4381–7. 15913299 10.1021/jf0502505

[27] Reis PMCL, Dariva C, Vieira GAB, et al Extraction and evaluation of antioxidant potential of the extracts obtained from tamarind seeds (*Tamarindus indica*), sweet variety. J Food Eng 2016;173:116–23.

[28] Novelli ELB, Diniz YS, Galhardi CM, et al Anthropometrical parameters and markers of obesity in rats. Lab Anim 2007;41:111–19. 17234057 10.1258/002367707779399518

[29] Committee for the Update of the Guide for the Care and Use of Laboratory Animals Guide for the Care and Use of Laboratory Animals. 8th ed; Institute for Laboratory Animal Research. Division on Earth and Life Studies; Washington: The National Academies Press; 2011:1–220.

[30] Bradford MM. A rapid and sensitive method for the quantitation of microgram quantities of protein utilizing the principle of protein-dye binding. Anal Biochem 1976;72:248–54. 942051 10.1016/0003-2697(76)90527-3

[31] Kakade M, Simons N, Liener I. An evaluation of natural vs. synthetic substrates for measuring the antitryptic activity of soybean samples. Cereal Chem 1969;46:518–26.

[32] Machado RJA, Monteiro NKV, Migliolo L, et al Characterization and pharmacological properties of a novel multifunctional Kunitz inhibitor from *Erythrina velutina* seeds. PLoS One 2013;38:e63571. 10.1371/journal.pone.0063571 PMC3666885 23737945

[33] Araújo JM, Alves JC, Peixoto TKON, et al Determinação da atividade antitríptica em proteínas de produtos do amendoim isoladas por cromatografia de afinidade. Quim Nova 2014;37:1618–23.

[34] Hall TA. BioEdit: a user-friendly biological sequence alignment editor and analysis program for Windows 95/98/NT. Nucleic Acids Symp Ser 1999;41:95–8.

[35] Gomes APG, Dias SC, Bloch C Jr, et al Toxicity to cotton boll weevil *Anthonomus grandis* of a trypsin inhibitor from chickpea seeds. Comp Biochem Physiol B Biochem Mol Biol 2005;140:313–19. 15649779 10.1016/j.cbpc.2004.10.013

[36] Dixon M, Webb EC. Enzyme inhibition and activation. New York: Academic Press; 1979;332–81.

[37] O’Farrell PH. High Resolution two-dimensional electrophoresis of proteins. J Biol Chem 1975;250:4007–21. 236308 PMC2874754

[38] Laemmli UK Cleavage of Structural Proteins during the assembly of the head of bacteriophage T4. Nature 1970;227:680–5. 5432063 10.1038/227680a0

[39] Davis DA, Soule EE, Davidoff KS, et al Activity of human immunodeficiency virus type 1 protease inhibitors against the initial autocleavage in Gag-Pol polyprotein processing. Antimicrob Agents Chemother 2012;56:3620–8. 22508308 10.1128/AAC.00055-12 PMC3393404

[40] Haq SK, Rabbani G, Ahmad E, et al Protease inhibitors: a panacea?. J Biochem Mol Toxicol 2010;24:270–7. 20135636 10.1002/jbt.20335

[41] Jedinák A, Maliar T. Inhibitors of proteases as anticancer drugs. Neoplasma 2005;52:185–92. 15875078

[42] Shamsi TN, Parveen R, Fatima S. Characterization, biomedical and agricultural applications of protease inhibitors: a review. Int J Biol Macromol 2016;91:1120–33. 26955746 10.1016/j.ijbiomac.2016.02.069

[43] Oliveira CFR, Vasconcelos IM, Aparicio R, et al Purification and biochemical properties of a Kunitz-type trypsin inhibitor from *Entada acaciifolia* (Benth.) seeds. Process Biochem 2012;47:929–35.

[44] Oddepally R, Sriram G, Guruprasad L. Purification and characterization of a stable Kunitz trypsin inhibitor from *Trigonella foenum-graecum* (fenugreek) seeds. Phytochemistry 2013;96:26–36. 24094275 10.1016/j.phytochem.2013.09.010

[45] Li X-Q, Andersson TB, Ahlström M, et al Comparison of inhibitory effects of the proton pump-inhibiting drugs omeprazole, esomeprazole, lansoprazole, pantoprazole and rabeprazole on human cytochrome P450 activities. Drug Metab Dispos 2004;32:821–7. 15258107 10.1124/dmd.32.8.821

[46] Fonseca FAH. Farmacocinética das estatinas. Arq Bras Cardiol 2005;85:9–14. 16400390

[47] Lingaraju MH, Gowda LR. A Kunitz trypsin inhibitor of *Entada scandens* seeds: another member with single disulfide bridge. Biochim Biophys Acta 2008;1784:850–5. 18359299 10.1016/j.bbapap.2008.02.013

[48] Moraes CS, Oliviera FOR, Masson G, et al Série em biologia celular e molecular: métodos experimentais no estudo de proteínas. Rio de Janeiro: IOC - Instituto Oswaldo Cruz; 2013:43–44.

[49] Gomes E, Guez MAU, Martin N, et al Enzimas termoestáveis: Fontes, produção e aplicação industrial. Quim Nova 2007;30:136–45.

[50] Cantú MD, Carrilho E, Wulff NA, et al Seqüenciamento de peptídeos usando espectrometria de massas: Um guia prático. Quim Nova 2008;31:669–75.

[51] Ostlund E, Yang W, Research L Relation between plasma leptin concentration and body fat, gender, diet, age, and metabolic covariates. J Clin Endocrinol Metab 1996;81:3909–13. 8923837 10.1210/jcem.81.11.8923837

[52] Maffei M, Halaas J, Ravussin E, et al Leptin levels in human and rodent: measurement of plasma leptin and ob RNA in obese and weight-reduced subjects. Nat Med 1995;1:1155–61. 7584987 10.1038/nm1195-1155

[53] Sáinz N, Barrenetxe J, Moreno-Aliaga MJ, et al Leptin resistance and diet-induced obesity: central and peripheral actions of leptin. Metabolism 2015;64:35–46. 25497342 10.1016/j.metabol.2014.10.015

[54] Meereis-Schwanke K, Klonowski-Stumpe H, Herberg L, et al Long-term effects of CCK-agonist and -antagonist on food intake and body weight in Zucker lean and obese rats. Peptides 1998;19:291–9. 9493861 10.1016/s0196-9781(97)00261-1

[55] Mhalhal TR, Washington MC, Newman K, et al Infusion of exogenous cholecystokinin-8, gastrin releasing peptide-29 and their combination reduce body weight in diet-induced obese male rats. Appetite 2016;109:172–81. 27916474 10.1016/j.appet.2016.12.001

[56] Crujeiras AB, Carreira MC, Cabia B, et al Leptin resistance in obesity: an epigenetic landscape. Life Sci 2015;140:57–63. 25998029 10.1016/j.lfs.2015.05.003

[57] Esser N, Legrand-Poels S, Piette J, et al Inflammation as a link between obesity, metabolic syndrome and type 2 diabetes. Diabetes Res Clin Pract 2014;105:141–50. 24798950 10.1016/j.diabres.2014.04.006

